# The Fas/Fas Ligand Death Receptor Pathway Contributes to Phenylalanine-Induced Apoptosis in Cortical Neurons

**DOI:** 10.1371/journal.pone.0071553

**Published:** 2013-08-07

**Authors:** Xiaodong Huang, Zhaohui Lu, Zhongwei Lv, Tingting Yu, Peirong Yang, Yongnian Shen, Yu Ding, Da Fu, Xiaoping Zhang, Qihua Fu, Yongguo Yu

**Affiliations:** 1 Department of Internal Medicine, Shanghai Children’s Medical Center, Shanghai Jiaotong University School of Medicine, Shanghai, China; 2 Department of Pediatric Cardiothoracic Surgery, Shanghai Children Medical Center, Shanghai Jiaotong University School of Medicine, Shanghai, China; 3 Department of Nuclear Medicine, Shanghai Tenth People’s Hospital, Tongji University School of Medicine, Shanghai, China; 4 Department of Laboratory Medicine, Shanghai Children Medical Center, Shanghai Jiaotong University School of Medicine, Shanghai, China; 5 Institute of Pediatric Translational Medicine, Shanghai Children Medical Center, Shanghai Jiaotong University School of Medicine, Shanghai, China; INSERM-Université Paris-Sud, France

## Abstract

Phenylketonuria (PKU), an autosomal recessive disorder of amino acid metabolism caused by mutations in the phenylalanine hydroxylase (PAH) gene, leads to childhood mental retardation by exposing neurons to cytotoxic levels of phenylalanine (Phe). A recent study showed that the mitochondria-mediated (intrinsic) apoptotic pathway is involved in Phe-induced apoptosis in cultured cortical neurons, but it is not known if the death receptor (extrinsic) apoptotic pathway and endoplasmic reticulum (ER) stress-associated apoptosis also contribute to neurodegeneration in PKU. To answer this question, we used specific inhibitors to block each apoptotic pathway in cortical neurons under neurotoxic levels of Phe. The caspase-8 inhibitor Z-IETD-FMK strongly attenuated apoptosis in Phe-treated neurons (0.9 mM, 18 h), suggesting involvement of the Fas receptor (FasR)-mediated cell death receptor pathway in Phe toxicity. In addition, Phe significantly increased cell surface Fas expression and formation of the Fas/FasL complex. Blocking Fas/FasL signaling using an anti-Fas antibody markedly inhibited apoptosis caused by Phe. In contrast, blocking the ER stress-induced cell death pathway with salubrinal had no effect on apoptosis in Phe-treated cortical neurons. These experiments demonstrate that the Fas death receptor pathway contributes to Phe-induced apoptosis and suggest that inhibition of the death receptor pathway may be a novel target for neuroprotection in PKU patients.

## Introduction

Phenylketonuria (PKU), one of the most common inborn errors of amino acid metabolism, leads to progressive mental retardation in children. Phenylketonuria is caused by mutations in the gene encoding the hepatic enzyme phenylalanine hydroxylase (PAH), which catalyzes the conversion of phenylalanine (Phe) to tyrosine. A deficiency in PAH activity leads to accumulation of Phe in brain tissue and cerebrospinal fluid, resulting in brain damage [1], [2]. Many of the neurodegenerative effects of PKU-associated Phe accumulation are indirect, including decreased creatine kinase activity, deficient myelin production, and reduced dopamine synthesis due to the lack of tyrosine [3]. In addition, however, high concentrations of Phe can trigger neuronal apoptosis directly [4].

There are two major pathways that lead to apoptosis [5]. One is the mitochondria-initiated intrinsic pathway, in which the release of cytochrome c from the mitochondrial matrix following loss of inner mitochondrial membrane integrity triggers formation of the apoptosome composed of Apaf-1, pro-caspase-9, dATP, and cytochrome c. Formation of the apoptosome leads to the activation of effector caspase-3, -6, and -7 [6], [7]. The other apoptotic pathway is the death receptor-initiated extrinsic pathway, in which death receptor ligation is followed by recruitment of adaptor molecules and activation of caspase-8 or caspase-10 [8], [9]. In addition, apoptosis can be induced via the endoplasmic reticulum (ER), which normally regulates protein synthesis and intracellular calcium (Ca^2+^) homeostasis. Excessive ER stress leads to increased cytosolic Ca^2+^ and ensuing activation of m-calpain. Activated m-calpain cleaves Bcl-xL and proteolytically activates caspase-12 [10], which then activates caspase-9 followed by activation of caspase-3 [11].

A recent study showed that a high concentration of Phe increased apoptosis in cultured neurons by activating the mitochondria-initiated intrinsic pathway [12]. Here we demonstrate that Phe can also trigger the death receptor-initiated extrinsic pathway in cultured cortical neurons.

## Materials and Methods

### Neuronal Culture

The use and care of animals followed the guidelines of the Shanghai Institutes for Biological Sciences Animal Research Advisory Committee and the study was approved by the Ethical Committee of Shanghai Children’s Medical Center. Sprague–Dawley rats were deeply anaesthetized by injection of sodium pentobarbital (100 mg/kg body weight). Primary rat cortical neurons were prepared from 14-day-old rat embryos as described [13]. Briefly, cortical neurons were plated on poly-d-lysine-coated dishes or coverslips and cultured in neurobasal medium (Gibco-BRL, Gaithersburg, MD) supplemented with 2% B27 (Gibco-BRL) and 0.5 mM glutamine (Gibco-BRL). Cytotoxicity experiments were performed on 3-day-old neuronal cultures. Cells were treated with 0.9 mM Phe for 18 h to induce apoptosis. For studies of mitochondria, cell death receptor, or ER-mediated apoptosis, cells were incubated with an apoptosis pathway inhibitor (either Z-VAD-FMK, Z-IETD-FMK, or salubrinal) and 0.9 mM Phe for 18 h.

### Chemicals and Reagents

Neurobasal medium and B27 were purchased from Gibco (St. Louis, MO, USA). Antibodies against FasL and cleaved caspase-3, -8, and -12 were purchased from Cell Signaling Technology (Beverly, MA, USA), and CD95-FITC was purchased from BD Biosciences (Mississauga, ON). The caspase-8-specific inhibitor Z-IETD-FMK was purchased from Calbiochem (San Diego, CA, USA). Horseradish peroxidase (HRP)-conjugated goat anti-rabbit IgG was purchased from Bio-Rad Laboratories (Richmond, CA). The In Situ Cell Death Detection Kit (Fluorescein) was purchased from Roche Molecular Biochemicals (Indianapolis, IN). The caspase inhibitor Z-VAD-FMK was purchased from Promega Biotech Co., Ltd (Madison, WI) and the eIF-2α inhibitor salubrinal (Sal) from Santa Cruz Biotechnology (Santa Cruz, CA, USA).

### TUNEL Assay

Fixed cells were permeabilized with 0.1% Triton X-100 in 0.1% sodium citrate.

Apoptosis was detected by TdT-mediated dUTP nick end labeling (TUNEL) using the in situ cell death fuorescence detection kit (Roche Molecular Biochemicals, Mannheim, Germany) according to the manufacturer’s instructions. DAPI was used as a nuclear counterstain to count total neurons. The intensity of TUNEL staining was examined by fluorescence microscopy and the specificity confirmed by morphological criteria such as condensation of chromatin and nuclear fragmentation. For quantification, apoptotic (TUNEL-positive) cells were counted in five randomly chosen fields from each treated culture. Cells were scored as positive (dark brown with blue nuclei) or negative (only blue nuclear DAPI staining) and apoptosis expressed relative to the total number of DAPI-stained cells.

### Western Blot Analysis

Caspase expression levels were estimated by Western blot analysis. Each treated culture was lysed in 0.3 mL lysis buffer (0.1% SDS, 1% NP-40, 50 mM HEPES [pH 7.4], 2 mM EDTA, 100 mM NaCl, 5 mM sodium orthovanadate, 40 mM p-nitrophenyl phosphate, and 1% protease inhibitor). The cell lysate was centrifuged at 12800×g for 25 min and then heated to 95°C for 5 min. Equal amounts of soluble protein in the supernatant were separated by 15% SDS-PAGE using actin as the loading control and then electroblotted onto nitrocellulose membranes (BioRad, CA). Membranes were incubated for 1 h in a blocking solution at room temperature (RT) and then overnight at 4°C in primary monoclonal antibodies. After five washed, the membranes were incubated in the appropriate horseradish peroxidase-conjugated secondary antibody (ECL, Amersham, England) for 1 h at RT. Luminescence was detected by X-ray film.

### Cell Preparation for Flow Cytometry (FCM)

Cultures were harvested and cell suspensions collected in 10 ml centrifuge tubes (at least 1×10^6^ cells/mL in each tube). The cells were precipitated by centrifugation (500−1000 r/min for 5 min), washed once by resuspension in the incubation buffer, and reprecipitated by centrifugation. After adding 2 µg/ml FITC-conjugated anti-CD95 (anti-FasR) or isotype-matched non-specific IgG (negative control) in the incubation buffer, cells were incubated in the dark at RT for 15 min, precipitated, washed once by resuspension in the incubation buffer, and then incubated with the fluorescent cell-labeling solution at 4°C for 20 min in the dark. Tubes were agitated frequently during labeling. After labeling, cells were washed with the incubation buffer, and the fraction of CD95-positive cells measured by FCM.

### Immunoprecipitation

Cells were collected by gentle scraping from the culture dish, washed twice in PBS, and lysed in 50 µL lysis buffer (20 mM Na_3_PO_4_ [pH 7.5], 500 mM NaCl, 0.1% SDS, 1% NP40, 0.5% sodium deoxycholate and 0.02% NaN_3_). The lysate was then incubated with an anti-CD95 antibody (sc-716; Santa Cruz Biotechnology) at 4°C with gentle rocking overnight. The immune complexes were collected by incubation with Protein G Agarose beads (Pierce) for 2 h. Beads were washed four times with lysis buffer, resuspended in Laemmli buffer, boiled for 10 min, and the released proteins resolved by SDS-polyacrylamide gel electrophoresis (Ready Gel; Bio-Rad Laboratories). Separated proteins were transferred to polyvinylidene difluoride (PVDF) membranes for immunoblotting. The presence of Fas was detected using the appropriate primary and secondary antibodies and band density measured by ECL.

### Statistical Analysis

All data are presented as mean ± SE of three or more independent experiments. Means were compared for statistical significance by one-way ANOVA. A value of P<0.05 was considered statistically significant.

## Results

### The ER Stress-initiated Pathway is not Involved in Phenylalanine-induced Apoptosis

**Figure 1:**
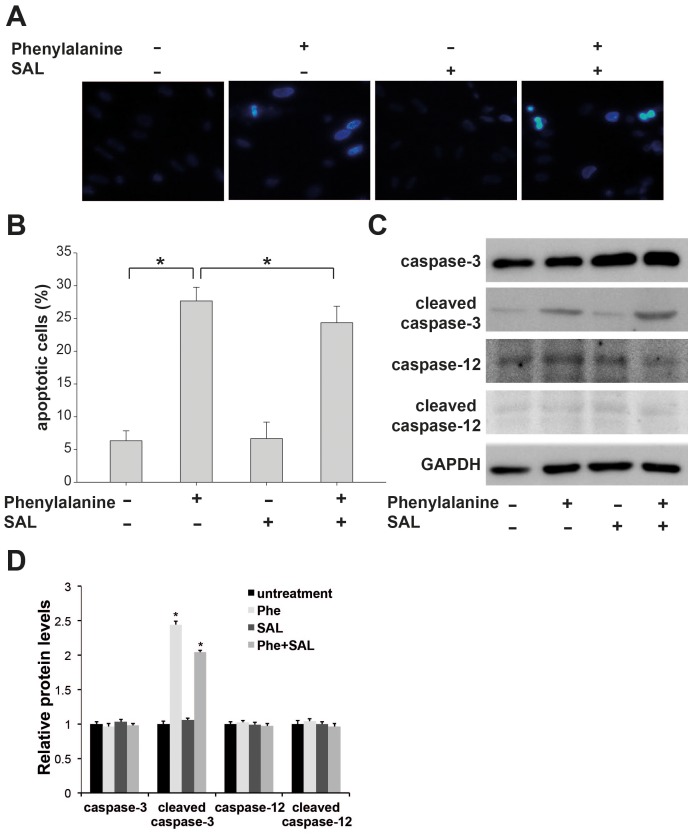
The ER stress-initiated pathway does not contribute to phenylalanine-induced apoptosis. (A) The eIF4 blocker salubrinal (SAL) did not block apoptosis induced by phenylalanine. Cells were pretreated with 100 ng/ml SAL prior to phenylalanine treatment (0.9 mM for 18 h). Apoptosis was measured by TUNEL assay. (B) Quantification of apoptosis induced by phenylalanine from (A). (C) SAL pretreatment did not affect activated caspase-3 or caspase-12 expression following phenylalanine treatment as measured by Western blotting. (D) Quantification of band intensity in (C). Values are mean ± SD of three independent experiments. * P<0.05.

To determine the apoptotic pathways that contribute to Phe-induced apoptosis, we first examined ER stress-induced apoptosis during Phe cytotoxicity. Many genetic diseases are caused by mutations that impair correct protein folding. In PKU, the mutant protein is efficiently degraded, however, and the disease arises instead from deficient Phe metabolism [14] [15]. Nonetheless, the PAH protein may induce ER stress and the unfolded protein response (UPR). The combination of this ER stress response and inherent Phe toxicity may initiate ER-mediated apoptosis. To test this hypothesis, we pretreated cortical neurons with Sal, an inhibitor of eIF2 dephosphorylation recently shown to counteract ER stress-induced cell death in cultured cells [16] and compared apoptosis to cultures treated with Phe alone. Western blot analysis was performed to determine the expression of full-length and cleaved (activated) caspase-3 and caspase-12 as an indicator of ER-mediated apoptosis. After 18 h of Phe treatment in the absence of Sal, neuronal nuclei were condensed and crescent-shaped (Fig. 1A), and there was a larger than 3-fold increase in the number of apoptotic cells compared to untreated cultures as indicated by TUNEL staining (Fig. 1B). The level of cleaved caspase-3 was significantly increased after Phe treatment (Fig. 1C, D), but there was no increase in caspase-12 activity (Fig. 1C, D). Pretreatment with Sal did not block apoptosis (Fig. 1A, B), and there was no change in the level of cleaved caspase-3 and caspase-12 following Phe treatment (Fig. 1B–D). These data confirm our previous observation that a high concentration of Phe can induce apoptosis in cortical neuronal cells. However, the ER stress-initiated pathway is not involved in this process.

### The Death Receptor Initiated Pathway is Involved in Phenylalanine-induced Apoptosis

**Figure 2:**
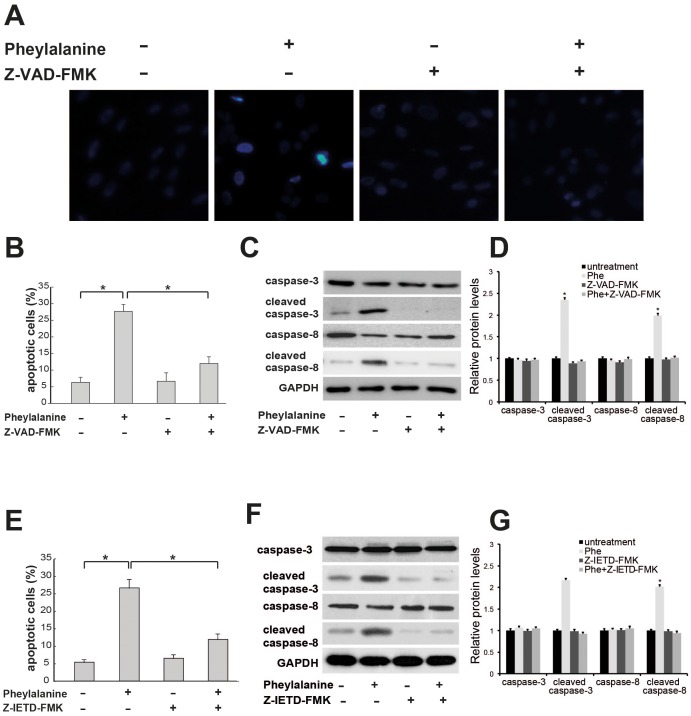
The death receptor-initiated pathway is involved in phenylalanine-induced apoptosis. (A) Phenylalanine-induced apoptosis is dependent on caspase activity. Cells were pretreated with 20 µM Z-VAD-FMK before phenylalanine (0.9 mM for 18 h) and apoptosis measured by TUNEL assay. (B) Quantification of apoptosis induced by phenylalanine from (A). (C) Z-VAD-FMK blocked caspase-3 and caspase-8 activation induced by phenylalanine. Cells were pretreated with 20 µM Z-VAD-FMK before phenylalanine treatment (0.9 mM for 18 h) and activated caspase-3 and caspase-8 activation measured by Western blotting. (D) Quantification of band intensity in (C). (E) Z-IETD-FMK blocked phenylalanine-induced apoptosis. Cells were pretreated with 20 µM Z-VAD-FMK before phenylalanine exposure as above. Apoptosis was measured by TUNEL assay. (F) Z-IETD-FMK blocked the phenylalanine-induced increase in activated caspase-3 and caspase-8 expression. (G) Quantification of band intensity in (F). Values are mean ± SD of three independent experiments. * P<0.05.

We next examined whether the death receptor-initiated pathway was involved in Phe-induced apoptosis. Execution of apoptosis requires the activation of caspase cascades [17] [18]. Typically, the death receptor-initiated pathway involves activation of initiator caspase-8, which in turn activates caspase-3 [9]. We pretreated cells with Z-VAD-FMK, a broad-spectrum caspase inhibitor [19], and compared the rate of apoptosis following Phe treatment (0.9 mM, 18 h) to cultures treated with Phe alone. Pretreatment with Z-VAD-FMK significantly decreased the number of apoptotic cells as measured by TUNEL staining (Fig. 2A, B). We next performed Western blot analysis to determine the expression level of caspase-8 and cleaved caspase-8 before and after Phe treatment alone and Phe following Z-VAD-FMK pretreatment. The level of cleaved caspase-8 increased significantly after Phe treatment (Fig. 2C, D) and Z-VAD-FMK pretreated significantly reduced both activated caspase-8 and caspase-3 expression (Fig. 2C, D). In addition, Z-VAD-FMK pretreatment significantly decreased the number of apoptotic cells following Phe exposure compared to cultures treated with Phe alone (Fig. 2B). To specifically block capase-8 activity, we pretreated the cells with Z-IETD-FMK [20]. Pretreatment with Z-IETD-FMK almost completely blocked the apoptosis induced by Phe (Fig. 2E) and completely inhibited the increase in caspase-8 and caspase-3 expression (Fig. 2F, G). These data strongly suggest that the death receptor-initiated pathway is involved in Phe-induced apoptosis.

### Fas/FasL Signaling Mediates Phenylalanine-induced Apoptosis

**Figure 3:**
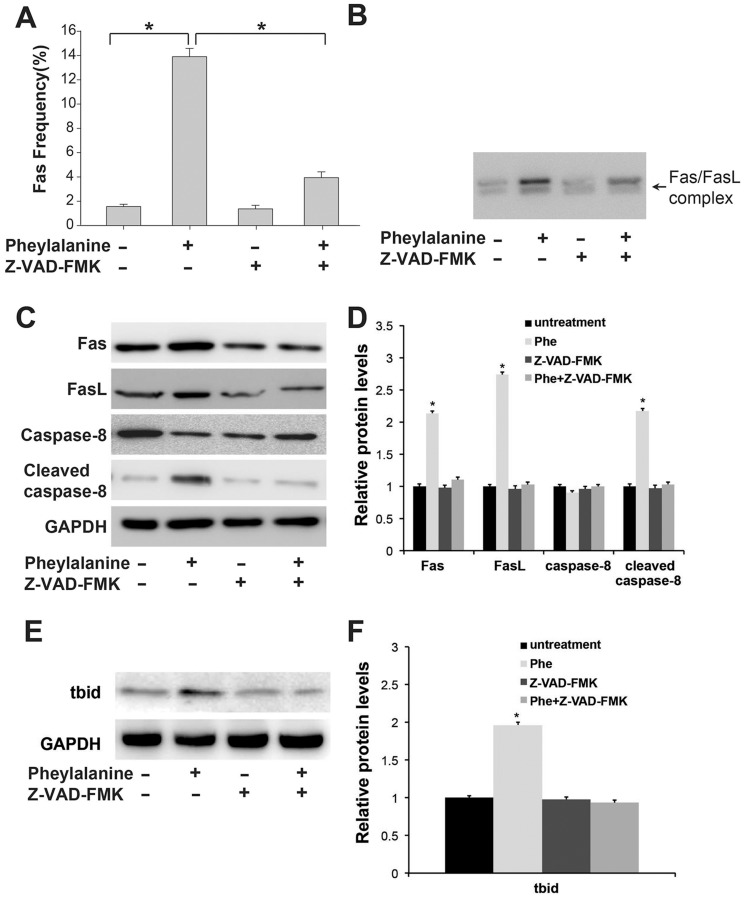
Fas/FasL signaling contributes to phenylalanine-induced apoptosis. (A) Cell surface Fas expression was upregulated by phenylalanine and reduced by Z-VAD-FMK pretreatment. Cells were pretreated with 20 µM Z-VAD-FMK before phenylalanine treatment (0.9 mM for 18 h) and cell surface of Fas expression was assessed by flow cytometry. (B) Dissociated Fas and FasL was decreased by phenylalanine and increased by Z-VAD-FMK pretreatment as assessed by immunoprecipitation. (C) Z-VAD-FMK blocked caspase-8 activation by phenylalanine. Caspase-3 and caspase-8 activation were determined by Western blot. (D) Quantification of band intensity in (C). (E) Z-VAD-FMK blocked Bidcleavage by phenylalanine. Expression of Bid was determined by Western blot. (F) Quantification of band intensity in (E). Values are mean ± SD of three independent experiments. * P<0.05.

The death receptor apoptotic pathway is activated by ligand-bound death receptors such as TNF-TNFR1, FasL-Fas, TRAIL-DR4, and TRAIL-DR5, of which the Fas/FasL pathway is perhaps the most ubiquitous and best characterized [9]. To determine whether Fas/FasL signaling is involved in Phe-induced apoptosis, we performed flow cytometry to measure cell surface expression of FasR (CD95) and immunoprecipitation to measure Fas/FasL complex formation. Indeed, Phe significantly increased cell surface Fas expression (Fig. 3A) and immunoprecipitation verified formation of the Fas/FasL complex after phenylalanine treatment, consistent with Phe-induced activation of the Fas pathway (Fig. 3B). Furthermore, pretreatment with the broad-spectrum caspase inhibitor Z-VAD-FMK prevented Phe-induced Fas upregulation (Fig. 3A, B). To investigate the downstream signaling events activated by Fas/FasL, the activation of caspase-8 and BID (a member of the Bcl-2 family of proteins) was assessed by Western blot analysis. Phenylalanine increased the level of cleaved caspase-8 and BID, while pretreatment with Z-VAD-FMK decreased the level of cleaved caspase-8 and BID following Phe treatment (Fig. 3C–F). These data show that the Fas/FasL-mediated pathway is activated during Phe-induced apoptosis.

### Blocking Fas/FasL Signaling Pathway Prevents Apoptosis Induced by Phenylalanine

**Figure 4:**
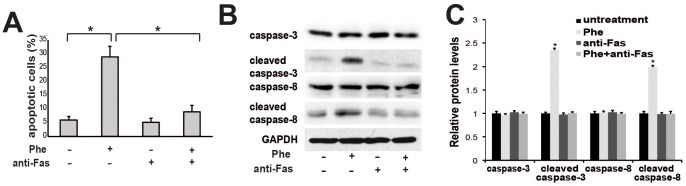
Blocking Fas/FasL signaling pathway prevents apoptosis induced by phenylalanine. Anti-Fas blocked phenylalanine-induced apoptosis. Cells were pretreated with 1 µM anti-Fas antibody 2 h before phenylalanine treatment (0.9 mM for 18 h) and apoptosis measured by TUNEL assay. (B) Anti-Fas antibody prevents caspase-8 and caspase-3 activation by phenylalanine. Caspase-3 and caspase-8 activation was determined by the Western blot. (C) Quantification of band intensity in (B). Values are mean ± SD of three independent experiments. * P<0.05.

To examine whether blocking Fas/FasL activation can prevent Phe-induced apoptosis, neurons were pre-incubated with a neutralizing antibody against Fas for 2 h and then treated with Phe. The anti-Fas antibody significantly decreased both the number of apoptotic cells (Fig. 4A) and the expression levels of cleaved caspase-8 and cleaved caspase-3 following Phe treatment (Fig. 4B, C).

## Discussion

Our previous studies demonstrated that high concentrations of phenylalanine (Phe) can increase apoptosis in cultured primary neurons, suggesting that direct Phe-mediate cytotoxicity contributes to neuronal injury in PKU [21]. Subsequent microarray studies indicated that the apoptosis-associated genes Bax, Bcl-x, and Bax were upregulated by Phe treatment [1]. A recent study also demonstrated the contribution of the mitochondria-mediated apoptotic pathway to Phe-induced death of cortical neurons. However, the contributions of other apoptotic pathways had not been investigated.

In this study, we show that the Fas/FasL death receptor-initiated extrinsic pathway also contributes to Phe-induced neuronal apoptosis. First, a high concentration of Phe (0.9 mM) upregulated cell surface expression of Fas, the formation of the Fas/FasL complex, and expression of caspase-3 and -8 (Fig. 3A, B; Fig. 2C, D). Second, both a broad-spectrum caspase inhibitor (Fig. 2B–D) and a specific caspase-8 inhibitor attenuated the Phe-induced increase in cell surface Fas expression, caspase-3 activation, and apoptosis (Fig. 2E–G). Finally, blocking Fas/FasL signaling using an anti-Fas antibody almost completely blocked caspase-3 activation, caspase-8 activation, and Phe-induced apoptosis (Fig. 4A–C). In contrast, Phe did not induce caspase-12 activation, indicating that ER stress is not responsible for Phe-induced apoptosis. Furthermore, blocking the ER pathway using the eIF2 inhibitor salubrinal did not reduce Phe-induced apoptosis or affect the expression of activated caspase-3 or caspase-12 (Fig. 1A–D).

Phenylketonuria is characterized by progressive neuronal loss, white matter abnormalities, dendritic retraction or failure of arborization, and reduced synaptic density. These neuropathological effects may be due to accumulation of high concentrations of Phe in the brain [22]. Our study provides a novel target for protecting neurons from cell death in PKU patients. It is well documented that the final steps in caspase-dependent apoptosis mediated by various signals are the activation of caspase-3 and ensuing caspase-3-mediated DNA fragmentation. Caspase-3 can be activated directly by caspase-8 through the death receptor pathway or by cleavage of the proapoptotic Bcl-2 family member protein BID. BID initiates the release of cytochrome c from mitochondria, thereby triggering the formation of the apoptosome complex that finally activates caspase-3 [6], [7], [8], [9]. Previous studies have demonstrated that Phe-induced apoptosis in cortical neurons serves as an excellent model system to study the mechanism of Phe neurotoxicity. Brain-derived growth factor (BDNF) partially protected neurons against Phe-induced apoptosis by interfering with the mitochondrial intrinsic apoptotic pathway [23]. Our results highlight the importance of the death receptor-initiated extinct pathway and suggest that blocking both pathways may be a more effective treatment strategy for protecting neurons in PKU patients. More importantly, our study also found that Fas/FasL signaling led to the cleavage of BID, which may contribute to activation of the mitochondria pathway.

In summary, we demonstrated a contribution of the Fas/FasL death receptor pathway in Phe-induced neuronal apoptosis. Preventing the upregulation of FasR expression or directly inhibiting the Fas/FasL pathway may be an effective therapeutic strategy for the prevention and treatment of brain injury in PKU patients.
